# Vinorelbine with or without Trastuzumab in Metastatic Breast Cancer: A Retrospective Single Institution Series

**DOI:** 10.1155/2014/289836

**Published:** 2014-03-30

**Authors:** Athina Stravodimou, Khalil Zaman, Ioannis A. Voutsadakis

**Affiliations:** ^1^Department of Oncology, University Hospital of Lausanne (CHUV), 1011 Lausanne, Switzerland; ^2^Division of Medical Oncology, Department of Internal Medicine, Sault Area Hospital, 750 Great Northern Road, Sault Ste Marie, ON, Canada P6B OA8

## Abstract

*Background*. We report our experience with vinorelbine, a widely used chemotherapeutic, in unselected metastatic breast cancer patients treated in clinical routine. *Patients and Methods*. The data of all patients with metastatic breast cancer receiving vinorelbine with or without trastuzumab during a six year period were reviewed. Patients received vinorelbine intravenous 25–30 mg/m^2^ or 60–80 mg/m^2^ orally in days 1 and 8 of a 21 day cycle. *Results*. Eighty-seven women were included. Sixty-two patients received vinorelbine alone and 25 patients received vinorelbine in combination with trastuzumab. In 67 patients this was the first line treatment for metastatic disease and in 20 patients it was 2nd or later line of treatment. The median TTP was six months (range: 1–45). The median overall survival was 11.5 months (range: 1–83). Seventy patients were evaluable for response. In patients receiving first line treatment 44.4% had a response while in the second and subsequent lines setting 12.5% of patients responded (*P* = 0.001). Objective response was obtained in 63.6% of patients receiving concomitant trastuzumab and in 25% of patients receiving vinorelbine alone (*P* = 0.0002). *Conclusion*. This study confirms a high disease control rate. Response rate and TTP were superior in first line treatment compared to subsequent lines.

## 1. Introduction

Breast carcinoma is the most common female cancer and among the most frequent causes of cancer mortality in women worldwide [1, 2]. Metastatic breast cancer is considered incurable with median survival estimates of about 2 to 3 years, but treatments with endocrine, cytotoxic, or targeted therapies can improve or maintain the quality of life and prolong survival.

Vinorelbine is one of the most widely used drugs in metastatic breast cancer. With the increasing use of anthracyclines and taxanes in the adjuvant setting there is a trend on advancing other drugs such as vinorelbine and capecitabine in earlier lines of treatment in the metastatic setting. Vinorelbine belongs to the family of vinca alkaloids together with vincristine, vinblastine, vindesine, and vinflunine [3]. All drugs of the vinca family share their mechanism of action, metabolism by hepatic disposition, and adverse effects profile. Vinorelbine differs from the older drugs of the family, vincristine and vinblastine, in that it is semisynthetic and has a higher affinity for tubulin and lipophilicity [3].

Progress in molecular biology has led to characterization of molecular subtypes of breast cancer based on genomic profiling. Surrogate clinically used subsets are characterized by the expression of a few proteins such as estrogen (ER) and progesterone (PR) receptors, EGFR family member protein Her2/Neu (HER2), and the Ki67 antigen as a marker of proliferation [4]. These markers can be reproducibly measured by immunohistochemistry (and/or an* in situ* hybridization method in the case of Her2) in clinical pathology laboratories and have become a standard component of breast cancer evaluation to define prognosis and help in therapeutic decisions. Breast cancer with HER2 protein overexpression or gene amplification represents a subset for which prognosis has been improved after the introduction of targeted therapies that inhibit the receptor [5]. The first of these therapies was the humanized monoclonal antibody trastuzumab registered more than 10 years ago. Trastuzumab has been shown to improve treatment efficacy both in the adjuvant and in the metastatic settings in combination with chemotherapy [5, 6]. Several chemotherapeutic drugs have been studied in HER2 overexpressing metastatic breast cancer in combination with trastuzumab including vinorelbine. In HER2 negative cancers vinorelbine is frequently used as monotherapy.

In this retrospective study we report retrospectively the experience of our center with vinorelbine (with or without trastuzumab) in unselected metastatic breast cancer patients in order to further define expectations in HER2 positive and negative disease and in the first and subsequent lines.

## 2. Patients and Methods

Data of patients included in this study were retrieved from our department patient archives containing the clinical data of women who received treatment for metastatic breast cancer. All patients with metastatic breast cancer receiving vinorelbine with or without trastuzumab during a six year period were retrospectively reviewed. Demographic data, biologic characteristics of the tumor and data on response, and time to progression (TTP) and overall survival (OS) were collected. Patients were treated with vinorelbine IV 25–30 mg/m^2^ or PO 60–80 mg/m^2^ in days 1 and 8 of a 21 day cycle. Patients with HER2 3+ tumors by immunohistochemistry or positive by FISH (Fluorescent* in situ* Hybridization) received trastuzumab in addition to vinorelbine while HER2 negative patients were treated with vinorelbine monotherapy. Patients who received vinorelbine in combination with other chemotherapeutics and patients with incomplete follow-up data were excluded. In patients that received concomitant trastuzumab a standard intravenous dosing schedule with 8 mg/kg loading dose followed by 6 mg/kg in subsequent administrations every three weeks was used.

Data collection and recording was conducted in compliance with the ethical requirements of our center and patients' anonymity was guaranteed. Due to the retrospective nature of the analysis and the fact that treatments had been provided according to standards of care, no specific informed consents were obtained.

Tumor response was recorded according to the revised RECIST (Response Evaluation Criteria in Solid Tumors) criteria [7]. Response rate was defined as the addition of complete response (CR) and partial response (PR). Disease control rate (DCR) was defined as the response rate plus the stable disease (SD) rate. TTP and OS were calculated by the Kaplan-Meier method starting from the date of vinorelbine therapy initiation to the date of disease progression documentation or death. Comparisons were made with the log rank test. The *x* ^2^ test was used to evaluate differences in clinical and biologic characteristics in the different groups. A Cox regression proportional hazard multivariate analysis was performed to identify statistically significant factors associated with TTP and OS. All *P* values were considered to be significant at the level of  *P* < 0.05. Statistical calculations were performed using an online tool from Denmark Technical University, Informatics Statistical Consulting Center (https://statcom.dk/K-M_plot.php) and a noncommercial statistical calculation site (http://www.statpages.org/).

## 3. Results

From 2006 to 2012, a total of 87 patients were fulfilling the criteria of the study. Baseline demographic and other characteristics of the included patients are presented in Table 1. The median age was 63 years (range: 32–85). Sixty-two patients received vinorelbine alone and 25 patients received vinorelbine with trastuzumab. In 67 patients this was the first line treatment for metastatic disease and in 20 patients it was 2nd or later line of treatment. Seventy patients were evaluable for response, while the remaining seventeen patients were not evaluable due to early progression (*n* = 6) or early termination of treatment for adverse effects (*n* = 11).

The overall response rate in evaluable patients in the cohort was 37.1% (1.4% complete response (CR) and 35.7% partial response (PR)). Eighteen additional patients (25.7%) had stable disease (SD) for at least three months resulting in a DCR of 62.8%. Twenty-four out of 54 patients (44.4%) receiving first line treatment obtained an objective response while in the second and subsequent lines of setting two of 16 patients (12.5%) responded (*x* ^2^ = 9.66, *P* = 0.001) (Table 2). 95% confidence intervals of different response groups are given in Table 2. DCR was 66.6% and 50% in patients in first and second or later line treatment, respectively. A response (CR or PR) was obtained in 63.6% of patients receiving concomitant trastuzumab and in 25% of patients receiving vinorelbine alone (*x* ^2^ = 13.63; *P* = 0.0002) (Table 3). DCR was 91% and 50% in patients receiving combined vinorelbine-trastuzumab and vinorelbine monotherapy, respectively. The response rate of patients with bone only metastases at the time of vinorelbine treatment was 30% and of patients with other sites of metastases (with or without bones) was 40% (*P* = 0.43).

The median TTP was six months (range: 1–45). Sixty-six patients of the cohort had died at the time of the analysis. The median overall survival was 11.5 months (range: 1–83). Seventy-three patients (83.9%) in the whole series had ER and/or PR positive and 14 (16.1%) had ER/PR negative breast cancer. Among evaluable for response patients, 60 (85.7%) had ER and/or PR positive cancers and had a response rate of 36.7% and 10 (14.3%) had ER/PR negative cancers and had a response rate of 40%. From the 48 HER2 negative patients who received vinorelbine alone only six were concomitantly ER and PR negative (triple negative). Four of them had PD, one SD and one PR. Adverse effects (mainly peripheral neuropathy and GI symptoms) necessitating interruption of treatment were observed in 18.5% of patients in the whole cohort.

**Figure 1:**
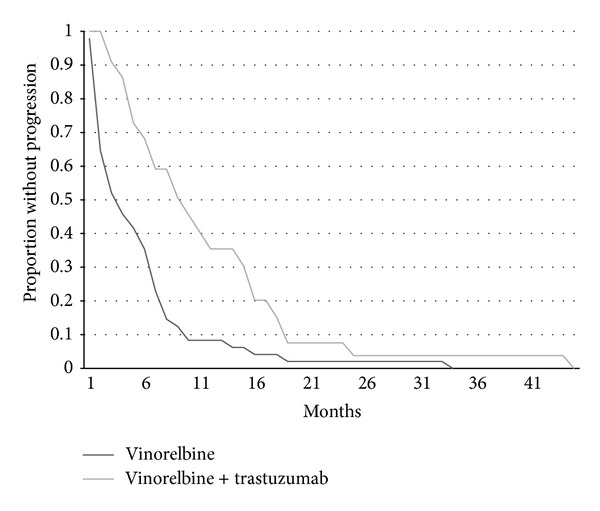
Kaplan-Meier curves of TTP for patients receiving vinorelbine alone (*n* = 48) or vinorelbine plus trastuzumab (*n* = 22). Log rank test *P* = 0.0015.

**Figure 2:**
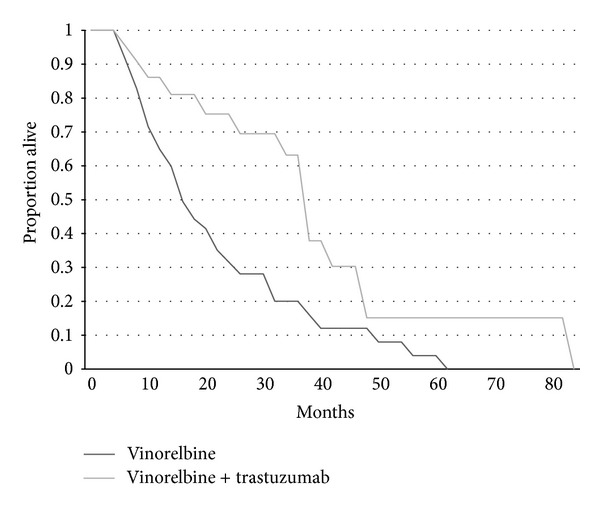
Kaplan-Meier curves of OS for patients receiving vinorelbine alone (*n* = 48) or vinorelbine plus trastuzumab (*n* = 22). Log rank test *P* = 0.0081.

**Figure 3:**
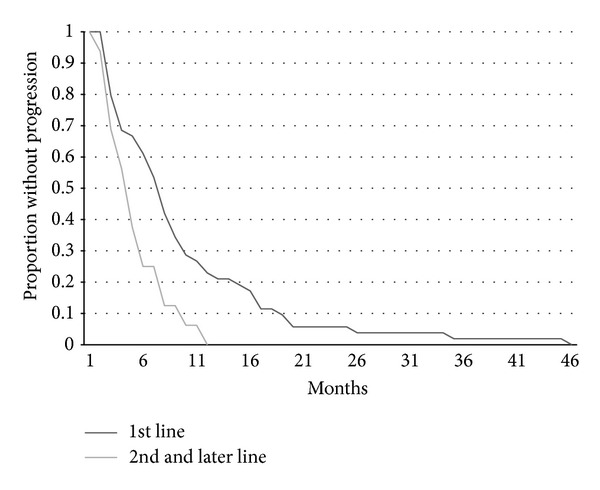
Kaplan-Meier curves of TTP for patients receiving vinorelbine with or without trastuzumab as 1st line metastatic treatment (*n* = 54) versus as 2nd or later line (*n* = 16). Log rank test *P* = 0.015.

**Figure 4:**
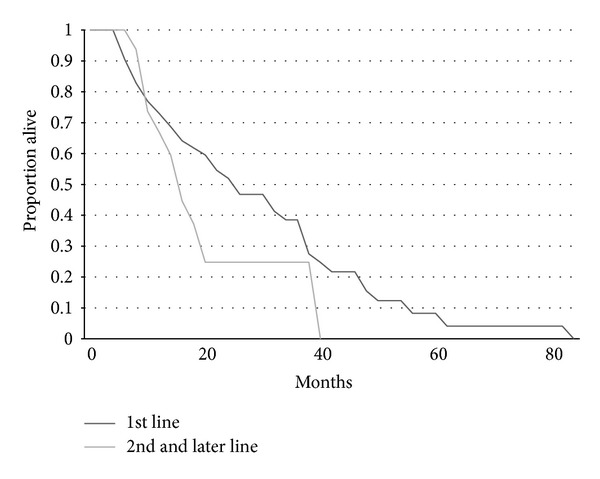
Kaplan-Meier curves of OS for patients receiving vinorelbine with or without trastuzumab as 1st line metastatic treatment (*n* = 54) versus as 2nd or later line (*n* = 16). Log rank test *P* = 0.14.

As expected both time to progression and overall survival were shorter for patients receiving vinorelbine alone compared with vinorelbine combined with trastuzumab (Figures 1 and 2). Time to progression of patients receiving vinorelbine-based treatment as first line metastatic therapy was also significantly longer than for patients that received it as second or later lines of therapy (Figure 3). Overall survival did not differ significantly according to whether patients received vinorelbine with or without trastuzumab as first line metastatic treatment or second and later line treatment (Figure 4).

Cox multivariate analysis showed that increasing age and absence of trastuzumab association in the regimen were statistically significantly associated with reduced overall survival, while ER/PR status and metastases location at the time of vinorelbine treatment (bone only versus other locations with or without bones) were not (Table 4). Similarly, second or later line of treatment was not associated with decreased OS, although approaching significance (*P* = 0.053). Regarding TTP, vinorelbine as second or later line of metastatic treatment and absence of trastuzumab association in the regimen were statistically significantly associated with shorter TTP, while age, ER/PR status, and metastases location at the time of vinorelbine treatment were not associated with TTP (Table 5).

## 4. Discussion

Metastatic disease is one of the greatest challenges faced by cancer specialists, partly because the variety of presentations, underlying biology, and increasing drug options make decisions regarding the optimal therapeutic approach complicated. Both combinations of drugs and sequential single-agent chemotherapy or endocrine therapy are reasonable options as first line therapy depending on the specific situation [8]. One should bear in mind that metastatic breast cancer therapy is not given with a curative intent and consequently respect of quality of life is one of the main goals of treatment. On the other hand a rapid and significant response to a treatment even with short term toxicities may be useful and valuable in case of high burden cancer (i.e., extensive visceral disease) [8].

One of the cytotoxics that are commonly used in metastatic breast cancer is vinorelbine [9, 10] particularly because of its good tolerance profile. Vinorelbine is a semisynthetic third generation vinca alkaloid that acts by inhibiting cancer cell proliferation. Vinca alkaloids are chemically constituted of a vindoline nucleus linked to a catharanthine moiety [3]. Their mechanism of action consists of interfering with mitotic spindle progression during cell cycle and blocking it during metaphase to anaphase transition. At higher concentrations, they stimulate microtubule depolymerization and promote mitotic spindle destruction. Their main molecular targets are tubulin and microtubules. Vinorelbine, similarly to other vinca alkaloids, binds to *β*-tubulin subunits at vinca-binding domain near the positive end of microtubules. This is the same binding site as that which binds alkaloid maytansine, the cytotoxic component of the recently introduced compound drug trastuzumab-DM. Taxanes and epipodophyllotoxins bind to a different *β*-tubulin domain. Vinorelbine binds rapidly and reversibly to soluble tubulin and induces a conformational change that increases the affinity of tubulin for itself. This change modulates the kinetics of microtubule stabilization, reduces the rate of microtubule dynamics (lengthening and shortening), and increases the duration which microtubules spend in an attenuated state. As a result the tension at the kinetochores of chromosomes during mitosis is reduced. Chromosomal condensation and separation along their length are observed but chromosomes lurk at the spindle poles and are unable to move properly towards the spindle equator [3]. Interruption of spindle dynamics triggers apoptosis that seems p53 independent [11]. Vinorelbine has distinct clinical advantages over other vinca drugs such as reduced peripheral neurotoxicity and oral availability. Its greater lipophilicity increases its concentrations in certain tissues such as lung compared with other drugs of the class [3, 12].

Several trials and retrospective series have explored the role of vinorelbine as monotherapy or with trastuzumab in metastatic breast cancer and some examples will be outlined below. In a phase III study of 252 anthracycline and taxanes pretreated patients, vinorelbine monotherapy was used as the control arm and compared with vinorelbine combined with gemcitabine [13]. The median PFS of the vinorelbine monotherapy arm was 4 months, the median OS was 16.4 months, and the response rate was 26%. Although the PFS was improved with the combination, the OS was not [13]. In a phase II trial of vinorelbine in 50 anthracycline and taxane pretreated patients the response rate was 20% and the TTP 115 days [14]. A recent small phase II study in patients previously treated with taxanes and anthracyclines showed a response rate to vinorelbine of 20.8% and a DCR of 58.3% [15]. The median TTP and OS were 3.7 and 10.4 months. No difference in response rate between the HER2 negative and HER2 positive patients, who did not receive trastuzumab in this study, was observed [15]. In another phase II study of oral vinorelbine monotherapy in the first line setting that included 64 patients, the overall response rate was 31% [16]. Median PFS was 17.4 weeks and median OS was not reached.

Responses observed with the combination of vinorelbine and trastuzumab in HER2 positive patients are in general higher than with monotherapy. A phase III trial that compared vinorelbine/trastuzumab with docetaxel/trastuzumab as later line treatment in HER2 positive patients pretreated with anthracyclines and taxanes showed an identical response rate of about 30% and 1 year survival of 88% in both arms [17]. The vinorelbine arm showed a longer TTP (15.3 versus 12.4 months) and was better tolerated than the docetaxel arm. In a phase II study of 42 patients that received vinorelbine and trastuzumab in the first line setting RR was 70%, median TTP was 9.3 months, and median OS was 35.6 months [18]. In another phase II study, patients had received no more than one previous line of therapy and their response rate was 50%, while median TTP and OS were 9.6 and 22.7 months, respectively [19]. A phase II trial in anthracycline and taxane pretreated patients showed a response rate of 30.3% and median TTP and OS of 6.8 and 12.4 months, respectively [20].

In a retrospective series in the first line treatment of HER2 positive disease, 67 patients treated with vinorelbine and trastuzumab obtained a 57% response rate which was inferior in this series to treatment with docetaxel and trastuzumab (77%) [21]. In the salvage setting the response rate of 60 HER2 positive patients treated with vinorelbine and trastuzumab was 28% [22]. In another series of 100 patients treated with oral vinorelbine with or without trastuzumab the response rate was 25% and the disease control rate 51% [23]. The response rate in first line and with trastuzumab was higher in a way similar to our patients. In a study of 68 patients with breast cancer, mostly (about 80%) treated in second or later line, 33 received vinorelbine alone and had an ORR of 27.3% and 35 received vinorelbine plus trastuzumab and had an ORR of 51.4% [24]. The TTP was 6 and 9 months and the OS 22 and 27 months, respectively. In a smaller series of 30 patients with HER2 positive metastatic breast cancer treated with oral vinorelbine (60 mg without escalation) and trastuzumab, the CR was 18%, PR 50%, and SD for more than 6 months 21% [25]. TTP was 9 months and both response rates and TTP were influenced by whether treatment was received as first or later line.

Our retrospective analysis confirms that vinorelbine-based therapy is a valid first line and salvage option for patients with metastatic breast cancer, in clinical situations where monotherapy is preferred. Vinorelbine effectiveness appears not to be modified by endocrine sensitivity of the tumor or metastases location. Nevertheless, as other therapies, this cytotoxic is less effective in patients who were pretreated with one or more lines of therapy for metastatic disease and better therapies are needed for these patients. The activity of trastuzumab combined with chemotherapy in HER2 overexpressing breast cancer has been documented and we have observed in the current cohort that the combination is highly effective in terms of TTP and OS. Introduction of alternative targeted therapies for HER2 negative patients to combine with vinorelbine or other chemotherapeutics that would improve efficacy without increasing toxicity is an urgent goal.

## Figures and Tables

**Table 1:** Characteristics of all patients in this series and of the subset that was evaluable for response to vinorelbine treatment.

	All patients *n* = 87 (%)	Evaluable for response patients *n* = 70 (%)
Age, median [range]	63 [32–85]	61 [32–85]
OS median [range]	16 months [5–83]	11.5 months [1–83]
Follow-up (patients alive)		
median [range]	13 months [3–50]	12 months [1–50]
Hormonal status		
ER/PR positive	73 (83.9)	60 (85.7)
ER/PR negative	14 (16.1)	10 (14.3)
Treatment		
Vinorelbine monotherapy	62 (71.3)	48 (68.6)
Vinorelbine + trastuzumab	25 (28.7)	22 (31.4)
Line of treatment		
1st line	67 (77)	54 (77.1)
2nd or later	20 (23)	16 (22.9)

**Table 2:** Clinical response rates of patients with metastatic breast cancer treated with vinorelbine (with or without trastuzumab) either as 1st or as later line of treatment.

	1st line; *n* = 54 (%)	95% CI (%)	2nd or later line; *n* = 16 (%)	95% CI
CR	1 (1.9%)	0–10.7	0	0–22.7
PR	23 (42.6%)	30.3–55.9	2 (12.5%)	2.2–37.3
SD	12 (22.2%)	13.0–35.1	6 (37.5%)	18.4–61.5
PD	18 (33.3%)	22.2–46.7	8 (50%)	28.0–72.0

CR: complete response, PR: partial response, SD: stable disease, PD: progressive disease, and 95% CI: 95% confidence interval.

**Table 3:** Clinical response rates of patients with metastatic breast cancer treated with vinorelbine either as monotherapy or with trastuzumab.

	VNR mono; *n* = 48 (%)	95% CI	VNR + TRZ; *n* = 22 (%)	95% CI
CR	0	0–8.8	1 (4.5%)	0–23.5
PR	12 (25%)	14.8–38.9	13 (59.1%)	38.7–76.8
SD	12 (25%)	14.8–38.9	6 (27.3%)	12.9–48.4
PD	25 (50%)	36.4–63.6	2 (9.1%)	1.3–29.0

CR: complete response, PR: partial response, SD: stable disease, PD: progressive disease, VNR mono: vinorelbine monotherapy, VNR + TRZ: vinorelbine and trastuzumab, and 95% CI: 95% confidence interval.

**Table 4:** Multivariate Cox regression model for possible factors influencing OS of patients with metastatic breast cancer. Age was included as a continuous variable. ER/PR status was considered positive if either of the receptors was positive and negative if both were negative. Line of treatment variable compares patients receiving vinorelbine containing treatment as first line metastatic therapy and those receiving it as second or later line. Sites of metastases variable compares patients with bone metastases only and those with other sites also present (with or without bones).

Variable	Hazard ratio	95% confidence interval		*P* value
		Lower limit	Upper limit	
Age	1.0275	1.0055	1.0499	0.014
ER/PR	1.1615	0.5982	2.2553	0.65
Trastuzumab	0.5032	0.2757	0.9183	0.025
Line of treatment	1.831	0.98	3.3891	0.053
Sites of metastases	1.1947	0.678	2.105	0.53

**Table 5:** Multivariate Cox regression model for possible factors influencing TTP of patients with metastatic breast cancer. Age was included as a continuous variable. ER/PR status was considered positive if either of the receptors was positive and negative if both were negative. Line of treatment variable compares patients who receive vinorelbine containing treatment as first line metastatic therapy and those receiving it as second or later line. Sites of metastases variable compare patients with bone metastases only and those with other sites also present.

Variable	Hazard ratio	95% confidence interval		*P* value
		Lower limit	Upper limit	
Age	1.0052	0.9866	1.0242	0.58
ER/PR	0.7171	0.3815	1.3479	0.30
Trastuzumab	0.4372	0.2557	0.7475	0.002
Line of treatment	1.9358	1.1168	3.3554	0.018
Sites of metastases	1.3463	0.8052	2.2511	0.25
